# Effect of piston texture at inclination and eccentricity work conditions on damping characteristics of a hydraulic shock absorber

**DOI:** 10.1038/s41598-022-13721-0

**Published:** 2022-06-13

**Authors:** Yangyang Yu, Junhong Zhang, Xiangde Meng, Dan Wang, Shasha Ma

**Affiliations:** 1 State Key Laboratory of Engine, Tianjin University, Tianjin, 300354 China; 2 Tianjin Renai College, Tianjin, 301636 China; 3 School of Civil Engineering, Tianjin University, Tianjin, 300354 China

**Keywords:** Engineering, Mechanical engineering

## Abstract

In order to accurately predict the damping characteristics of a hydraulic shock absorber under piston inclination and eccentricity conditions, especially, considering effects of piston surface construction. In present work, taking account of piston slight inclination and eccentricity, a more detailed mathematical model was developed to estimate effects of piston texture on damping characteristics. Based on the mathematical models of reservoir and compression stroke coupled with Reynolds equation, a new damping force model was developed, which analyzed effects of piston structure on damping characteristics. The mathematical models of piston texture, piston slight inclination, piston eccentricity and combinations of three cases are developed to analyzed in detailed effects of piston texture at different work conditions on damping characteristics. The results shown that the friction force of piston increases parabolically with increasing depth ratio, and that of piston increases linearly with increasing area ratio. Piston textures have little effects on damping characteristics at specific structural parameters conditions when piston normal operation, however, textures of slight inclined and eccentric piston have great effects. As a result, piston textures might cause high damping force, destroyed comfort and safety. Therefore, it is necessary that effects of piston surface construction were precisely predicted on damping characteristics under different work conditions. The results might provide a new insight for the design of hydraulic shock absorber and investigation of vehicle system dynamics.

## Introduction

Dual-tube hydraulic shock absorber has been widely used in automobile suspension and railway vehicle suspension system^1,2^ since the mature technology and moderate cost. The contemporary age, as for automobile and railway vehicle, comfort and safety are paid special attention while pursuing higher velocity. Dynamic damping characteristics of the hydraulic shock absorber have great impact on the dynamic performance of vehicles^3,4^. Dynamic damping characteristics depend on the structure of the shock absorber. However, the structures are designed by the traditional design method including experience, then revised and adjusted by repeating experiments. It will take a long period and high cost. The structures are also designed by numerical simulation method. It is precise, fast, and convenient. However, the shock absorber usually works under complex conditions. It is difficult to obtain precisely damping performance at different complex work conditions. Therefore, it is an important research focus to precisely predict the performance of shock absorber by numerical simulation method to design optimal structures suitable for complex working conditions.

Structures of the shock absorber have great impact on dynamic damping characteristics. Duym^5,6^ and Yung^7^ established detailed modeling including the internal structure and operation process, analyzed shock absorption performance on internal structure. Besinger^8^, Berger^9^ and Lion^10^ established rheological model including buffer, spring and friction, and analyzed the effect of structural parameters on the damping characteristics. Czop^11^ formulated, derived and validated the first-principle non-linear model, investigated structural vibrations on the dynamical interactions between mounting elements, valve systems and the hydraulic actuator of the shock absorber, and captured dynamical characteristic on a wide operating range. Zhang^12^ elaborated the working principle of membranous dual-cavity based on amplitude sensitive damper (MASD), its dynamic model was derived by combining first-principle modeling of hydraulic components and empirical modeling of membranous valve. At the same time, the influence of the piston and pattern valve construction was analyzed on the dynamic damping. Alireza Farjoud^13^ presented nonlinear model of monotube hydraulic dampers, and emphasis on detailed structure of shim stack and their effects on the overall damper performance. Zhou^14^ established mechanics model of flexible ring throttle-slice based on elastic mechanical principals. The effect of superposition throttle-slices thickness on throttle opening size was researched in depth. Wang^15^ established a new full-parameter model and revealed the nonlinear displacement dependent characteristics of a high-speed rail pantograph dampers. The damping characteristics are analyzed on the internal cross-sections and dimensions of the orifices in the rod by the full-parameter model. Farfan-Cabrera^16^ contributed review on the current state and future improvement trends for optimization of critical tribological components which used in vehicles, gave an understanding of the most recent achievements in terms of tribological solutions applied to the critical components. The friction between the piston and cylinder of the hydraulic shock absorber has a critical effect on damping characteristics of the shock absorber, which provides an important direction for the finer and more comprehensive modeling of the shock absorber. Ji^17^ and Zhang^18^ set up a model on the damping force which considering the friction between the piston and cylinder, analyzed the damping performance of shock absorber. But friction was calculated by a constant or empirical formula, which could not completely reflect the effect of piston structure (including piston surface morphology) on the damping characteristics of the shock absorber.

However, it is lubrication contact between the piston and cylinder of the shock absorber. the effect of surface structures is crucial to the friction performance of lubrication contacts. Especially, the Reynolds equation is widely used to solve friction analysis of the hydrodynamic sliding bearing^19^, the piston and cylinder of engine and hydraulic cylinder^20–22^, and effect of bearing surface and piston structure could be analyzed on friction in detailed. The condition and principle of dynamic lubrication between piston and cylinder of double-cylinder hydraulic shock absorber are the same as that of dynamic sliding bearing, piston and cylinder of engine and hydraulic cylinder. With the rapid development of vehicle and train, the velocity increases and become higher and higher, the comfort of vehicle and train system becomes more sensitive to parameter variations of component, especially effect of friction on piston surface. Thus, it is imperative to establish more accurate models on the damping characteristics to investigate effect of piston surface structure.

The work conditions of automobile and railway vehicle suspension system are complex and varied. The inclination and eccentricity of the piston are frequently caused under long-term and high-velocity operation, and have great effects on the damping characteristics, causing high damping force to destroy comfort and safety. Wang^23^ addressed a more subtle and comprehensive non-linear parametric model of a highspeed rail hydraulic yaw damper, which accurate and robust predicted the damping characteristics with extremely wide velocity range. Alonso^24^ dealt with the modelling of yaw dampers and determined the influence of the modelling of this component on the obtained results when predicting the dynamic stability of a vehicle. It was verified that the accurate modelling of the yaw damper is critical when dealing with the vehicle s dynamic performance. Huang^25^ set up simplified model of the yaw damper, analyzed its dynamic performance in the range of operating conditions, concluded that the great difference between dynamic and static conditions was caused by the internal damper flexibility under small amplitudes, causing the breakdown occurrence at special working condition or the long-term work. Sun^26^ studied distortion characteristics of the shock absorber based on the energy method, obtained that the anti-distortion ability of the shock absorber increases with increasing inflation pressure. The wedge gap between piston and cylinder is generated by inclined and eccentric piston under operating, which caused large friction. Especially, piston surface structure also has great effect on the shape of wedge gap under piston inclination and eccentricity condition, thereby affecting damping characteristics. However, the effects of piston texture on damping characteristics of hydraulic shock absorber under different work conditions are very crucial, which has not been investigated in detail.

In order to investigate the effects of piston surface structure on damping characteristics of shock absorber when the piston is inclined or eccentric, in present work, taking account of piston slight inclination and eccentricity, a more detailed mathematical model is developed to estimate the effect of piston texture on dynamic damping characteristics of shock absorbers. The current work demonstrates the following new contributions: (1) Based on the mathematical models of reservoir and compression stroke coupled with Reynolds equation, a new damping force model was developed, which analyzed effects of piston surface structure on damping characteristics. (2) Damping force was analyzed in detailed under different work conditions including piston texture, piston eccentricity with texture, piston inclination with texture and piston eccentricity plus inclination with texture. (3) With increasing depth ratio *δ* and area ratio S_p_ of piston texture, the increasing form of the friction force was investigated. The effect of cylindrical texture on friction force at piston normal operation or inclined and eccentric piston conditions was analyzed. As a result, the results of this study might provide a new insight for the design of hydraulic shock absorber and investigation of vehicle system dynamics.

## Simulation model

**Figure 1:**
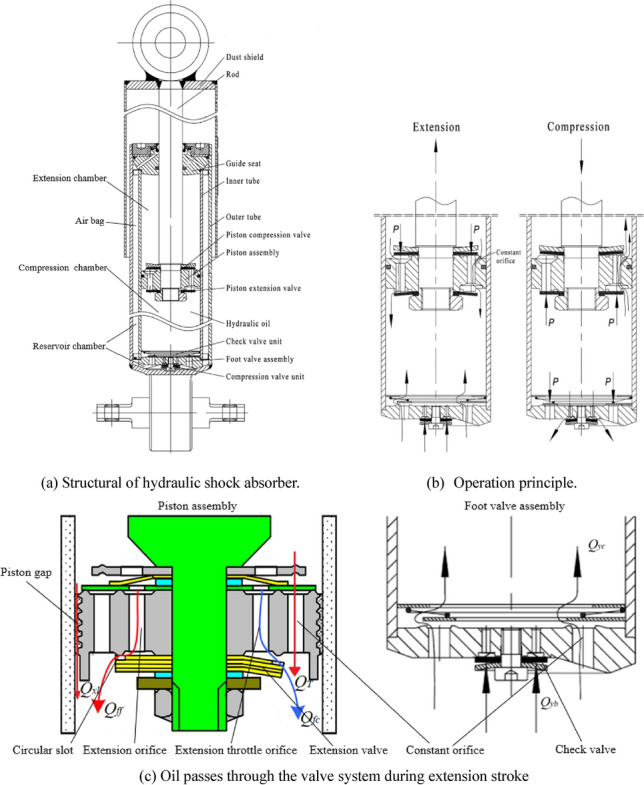
Hydraulic shock absorber.

Figure 1 shows the structural sketch of the double-cylinder hydraulic shock absorber, reflects the work process of extension stroke and compression stroke. The oil passes through the valve system during extension stroke and compression stroke, which produces damping force and reduces vibration energy. The double-cylinder hydraulic shock absorber absorbs the vibration energy.

### Extension and compression stroke

The oil passes through the constant orifice and piston gap when the extension valve is not opened. *Q*_T_ is the flow rate when the oil passes through the constant orifice of piston assembly. *Q*_*xl*_ is the flow rate when the oil passes through the piston gap. *Q*_T_ and *Q*_*xl*_ are expressed as follows:

1 $$Q_{T} = C_{q} A_{T} \sqrt {\frac{{2(P_{1} - P_{2} )}}{\rho }}$$

2 $$Q_{xl} = \frac{{2.5\pi d_{h} h^{3} (P_{1} - P_{2} )}}{{12\mu L_{y} }}$$

where *C*_*q*_ is flow coefficient of the constant orifice of piston assembly, *A*_*T*_ is total area of the constant orifice of piston assembly; $\rho$ is oil density, *d*_*h*_ is piston diameter; $\mu$ is dynamic viscosity of the oil; *L*_*y*_ is axial width of piston; *h* is actual oil film thickness between piston and cylinder; *P*_1_ is the pressure of extension chamber and *P*_2_ is the pressure of compression chamber.

The total flow *Q*_*fh*_ of oil from the extension chamber into the compression chamber is expressed as follows:

3 $$Q_{fh} { = }Q_{T} + Q_{xl}$$

Total flow rate *Q*_*fh*_ includes flow rate *Q*_*T*_ of the constant orifice of piston assembly, flow rate *Q*_*xl*_ of the piston gap and flow rate *Q*_*f*_ of the extension orifice when the extension valve is opened. As shown in Fig. 1c, flow rate *Q*_*f*_ of the extension orifice includes flow rate *Q*_*fc*_ of the extension throttle orifice and flow rate *Q*_*ff*_ of the circular slot. *Q*_*fc*_ and *Q*_*ff*_ are in series, thus, *Q*_*fc*_ = *Q*_*ff*_.

4 $$Q_{fc} = \varepsilon_{fc} A_{fc} \sqrt {\frac{{2(P_{1} - P_{2} )}}{\rho }}$$

5 $$Q_{ff} = \frac{{\pi \delta_{rf}^{3} (P_{1} - P_{2} )}}{{6\mu \ln (r_{bf} /r_{kf} )}}$$

where $\varepsilon_{fc}$ is the flow coefficient of extension throttle orifice, *A*_*fc*_ is the total area of extension valve orifice, *r*_*bf*_ is the outside radius of extension valve plate and *r*_*kf*_ is notch radius of the extension valve plate. $\delta_{rf} = f_{rf} - f_{rf0}$,$f_{rf}$ is deformation of extension valve and $f_{rf0}$ is pre-deformation of extension valve.

Flow rate *Q*_*yc*_ of the constant orifice of foot valve assembly and flow rate *Q*_*yb*_ of check valve are expressed as follow:

6 $$Q_{yc} = \varepsilon_{yc} A_{yc} \sqrt {\frac{{2(P_{3} - P_{2} )}}{\rho }}$$

7 $$Q_{yb} = \frac{{\pi \delta_{yb}^{3} (P_{3} - P_{2} )}}{{6\mu \ln (r_{bb} /r_{kb} )}}$$

where $\varepsilon_{yc}$ is flow coefficient of the constant orifice of foot valve assembly, *A*_*yc*_ is the total area of the constant orifice of foot valve assembly, *P*_3_ is pressure in reservoir chamber, *r*_*bb*_ is outside radius of check valve plate and *r*_*kb*_ is notch radius of check valve plate.$\delta_{yb} { = }f_{ry} - f_{ry0}$, $f_{ry}$ is deformation of check valve and $f_{ry0}$ is pro-deformation of check valve.

Deformation of valve plate is expressed as follows:

8 $$f_{rf} = \frac{\Delta P}{{h_{ffp} }}G_{rffp}$$

where *h*_*ffp*_ is the thickness of valve plate and *G*_*rffp*_ is the coefficient of valve plate deformation.

**Figure 2:**
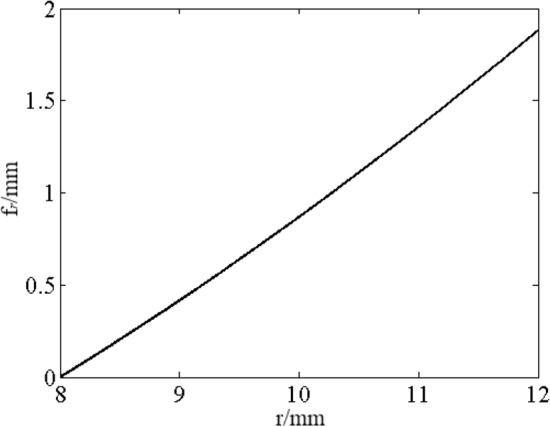
Deformation curve of valve plate.

Bending deflection of circular disc at arbitrary radius *r*^14^ is expressed as follows, and as shown in Fig. 2.

9 $$f_{r} = \frac{P}{{h^{3} }}G_{r}$$

10 $$G_{r} = T_{c1} \ln r + T_{c2} r^{2} \ln r + T_{c3} r^{2} + T_{c4} + T_{B} r^{4}$$

When the extension valve is opened, the total flow rate *Q*_*fh*_ of oil from the extension chamber into the compression chamber is expressed as follows:

11 $$Q_{fh} = Q_{T} + Q_{f} { + }Q_{xl}$$

Total flow rate *Q*_*yd*_ of oil from reservoir chamber to compression chamber is expressed as follows:

12 $$Q_{yd} = Q_{yc} + Q_{yb}$$

Assuming the gas of the reservoir chamber is ideal gas, and the expression can be given as:

13 $$P_{3} (t)V(t) = P_{30} V_{0}$$

14 $$V(t) = V_{0} + YA_{g}$$

where *V*_*0*_ is initial volume of the gases in the reservoir chamber, *P*_30_ is initial pressure of the gases in the reservoir chamber, *V*(t) is the volume of the gases in the reservoir chamber, *Y* is the relative displacement of piston and *A*_g_ is the section area of piston rod.

Assuming sinusoidal excitation is applied to the shock absorber, the relative displacement of the piston is expressed as follows:

15 $$\left\{ {\begin{array}{*{20}c} {Y = Y_{0} \sin (\omega t)\;{\kern 1pt} {\kern 1pt} } \\ {U = \omega Y_{0} \cos (\omega t)} \\ \end{array} } \right.$$

The compression stroke is similar to the extension stroke, thus work of compression stroke is not duplicated. The extension chamber pressure *P*_1_, the compression chamber pressure *P*_2_ and the reservoir chamber pressure *P*_3_ are obtained by relationship (13) between oil flow rate passing through the piston assembly and foot valve assembly and piston velocity during extension stroke and compression stroke.

16 $$\left\{ {\begin{array}{*{20}c} {Q_{fh} = U(A_{h} - A_{g} )} \\ {Q_{yh} = U(A_{h} - A_{g} )} \\ {Q_{yd} = UA_{g} \quad \quad \quad } \\ \end{array} } \right.$$

where *U* is the piston velocity, *A*_*h*_ is cross section area of piston and *Q*_*yh*_ is the total flow of oil from the compression chamber into the extension chamber.

### Piston/cylinder friction pair lubrication control equation of shock absorber

For a piston of shock absorber under stable working conditions, the two-dimensional Reynolds equation can be expressed in the following form^19^:

17 $$\frac{\partial }{\partial x}\left( {\frac{{h^{3} }}{\mu }\frac{\partial p}{{\partial x}}} \right) + \frac{\partial }{{\partial {\text{y}}}}\left( {\frac{{h^{3} }}{\mu }\frac{\partial p}{{\partial y}}} \right) = 6U\frac{\partial h}{{\partial x}} + 6\frac{\partial h}{{\partial t}}$$

where *p* is the oil film pressure at a specific point on the piston surface.

#### Actual oil film thickness

The expression of actual oil film thickness *h* between piston and cylinder can be obtained as follows:

18 $$h = h_{0} + \sum\limits_{i} {h_{pi} } \;\;\left( {i\, = \,{1},{2},{3}} \right)$$

where *h*_0_ is initial oil film thickness between piston and cylinder and *h*_*pi*_ is oil film thickness on outer surface of piston under different cases.

**Figure 3:**
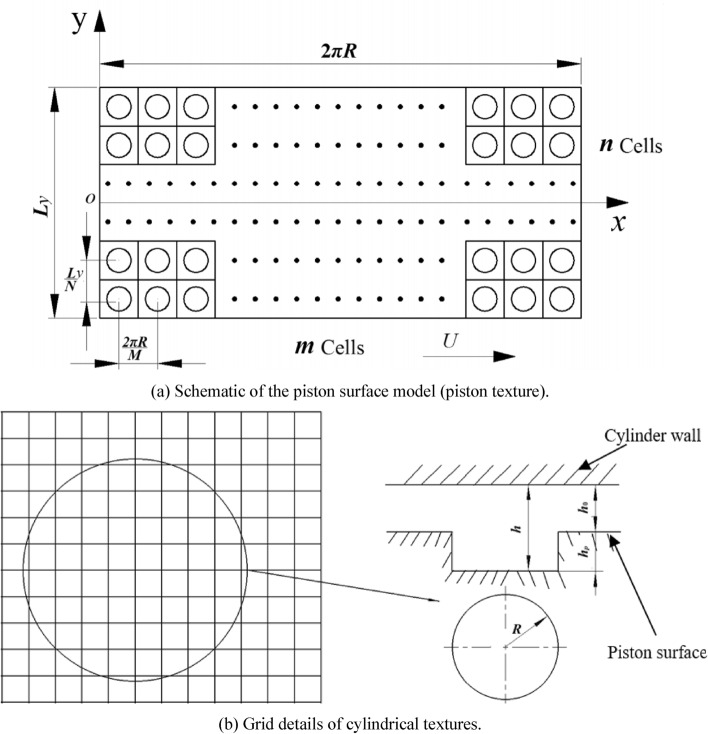
Schematic of piston texture.

**Figure 4:**
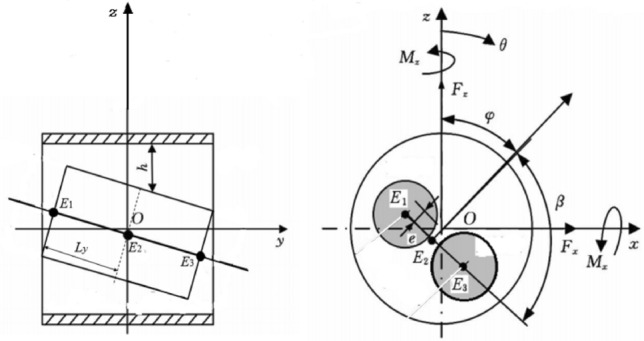
Schematic piston eccentricity and inclination.

**Figure 5:**
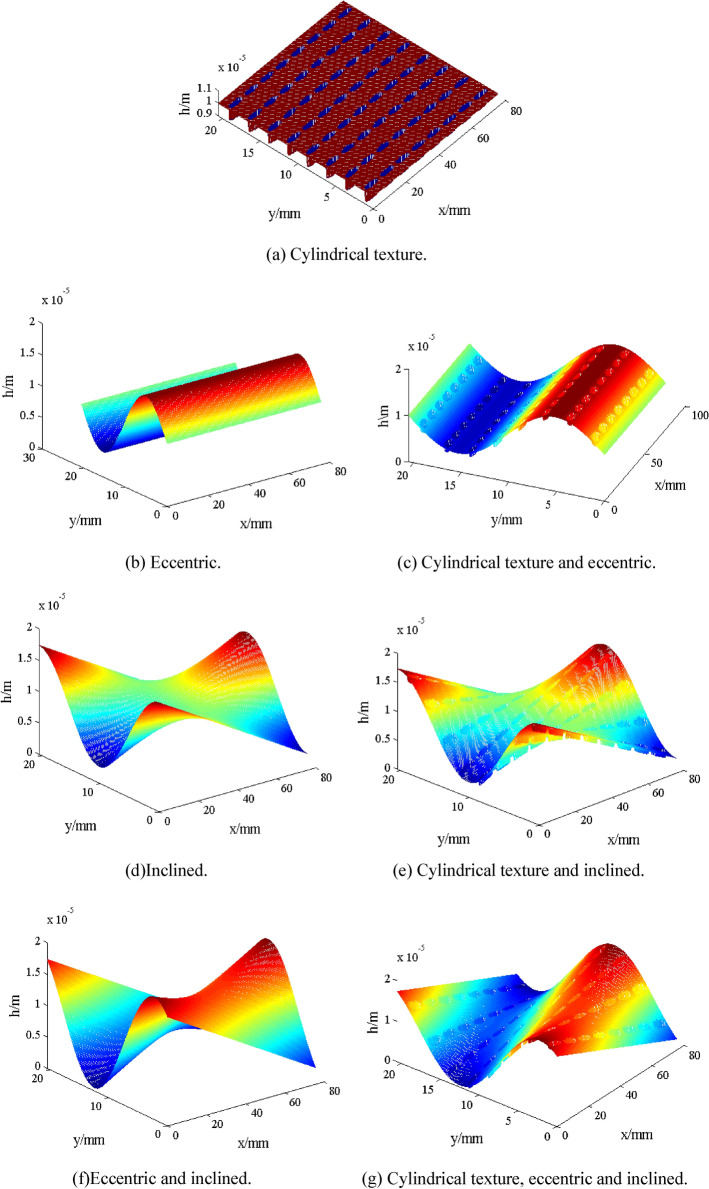
Distribution of oil film thickness on the outer surface of the piston under different cases.

Piston surface has regular shallow texture since machining tolerance and machining precision. Assuming the cylindrical textures are evenly distributed over the piston surface. Figure 3a,b shows schematic of piston textures. Considering piston eccentricity and inclination, Fig. 4 shows schematic of piston textures and the schematic of piston eccentricity and inclination. Figure 5 shows distribution of oil film thickness on the outer surface of the piston under different cases: cylindrical texture (Fig. 5a), piston eccentricity vs. piston eccentricity with texture (Fig. 5b vs. Fig. 5c), piston inclination vs. piston inclination with texture (Fig. 5d vs. Fig. 5e), and piston eccentricity plus inclination vs. piston eccentricity plus inclination with texture (Fig. 5f vs. Fig. 5g). Their oil film thickness *h*_*pi*_^21,27^ under different cases are expressed as follows:

19a $$h_{p1} = \left\{ {\begin{array}{*{20}c} {0\quad {\kern 1pt} {\kern 1pt} x^{2} + y^{2} \le r_{p}^{2} } \\ {c_{0} \quad x^{2} + y^{2} \le r_{p}^{2} } \\ \end{array} } \right.$$

19b $$h_{p2} = e\cos (\theta - \varphi )$$

19c $$h_{p3} = \tan \gamma \left( {y - \frac{{L_{y} }}{2}} \right)\cos (\theta - \beta - \varphi )$$

where *e* is the eccentricity of central section of piston, *θ* is the angular coordinate starting from the z axis, *φ* is the angle between *OE*_2_ and the *z* axis, *γ* is angle of the piston inclination and *β* is the angle between *OE*_2_ and *E*_1_*E*_3_.

#### Boundary conditions

The flow field between piston and cylinder is a convergent lubrication gap, the Reynolds boundary is applied in the piston modelling process. The effect of cavitation is not included in the analysis, either in terms of the single-phase analysis used or in the application of the boundary conditions, which is considered in the analysis of the piston^20,21^. *P*_0_ is the atmosphere pressure. The boundary conditions are expressed as follows:

20 $$p(x,y = 0) = P_{1} ,\;\;\;p(x,y = L_{y} ) = P_{2} ,\;\;p(x = 0,y) = P_{0} ,\;\;p(x = 2\pi R,y) = P_{0}$$

#### A set of assumptions

The work process of hydraulic shock absorber is a complex nonlinear fluid system. Damping characteristics of the shock absorber are affected by many factors including temperature, oil properties and assembly accuracy of each component, and so on. Some factors are ignored in the detailed mathematical model of investigating damping characteristics, including oil temperature, cavitation, gas, oil compressibility, oil density variation. A set of assumptions are shown as follow:

1. Assuming the solubility of the gas in the oil is ignored, gas does not dissolve in oil. Cavitation also is ignored.
2. Assuming oil temperature is fully dissipated during the shock absorber operation. Oil temperature remains constant. Temperature variation characteristics of oil are ignored. Oil viscosity remains constant. The environment temperature is 20 °C. Assuming the oil temperature and environment temperature are same.
3. Assuming the oil is incompressible. The oil will not vaporize due to temperature.
4. Assuming the gas of the reservoir chamber is ideal gas, its pressure and volume change in accordance with the laws of thermodynamics.
5. Assuming the oil pressure in each work chamber of the shock absorber is equal, and the pressure varies continuously with the reciprocating motion of the piston in the chamber.
6. All parts of the shock absorber are well assembled.

#### Numerical solution

In the *x*O*y* plane, the piston surface is meshed into *m* and *n* grids along the *x* and *y* directions. The five-point difference method is used to discrete Eq. (17). The symmetric successive over relaxation (SSOR) method is used to solve the discrete algebraic equation and the pressure *p* is obtained.

1. Load capacity
  The calculated oil film pressure *p* is numerically integrated in the whole fluid domain along the *x* and *y* directions, and the load capacities *W*_*N*_ can be obtained as follows:
  21 $$W_{N} = \int_{0}^{{L_{x} }} {\int_{0}^{2\pi R} p } dxdy$$
2. Friction force
  The calculation of the friction force on the piston is as follows:
  22 $$F_{foil} = \iint\limits_{S} {\left(\frac{h}{2}\frac{\partial p}{{\partial x}} + \frac{\mu U}{h}\right){\text{d}}x{\text{d}}y}$$

### Mathematical model of damping force

The dynamic damping characteristics of the shock absorber are mainly determined by the damping force *F*_*f*_: The damping force *F*_*f*_ is expressed as follows:

23 $$F_{f} = P_{1} (A_{h} - A_{g} ) - P_{2} A_{h} + F_{foil}$$

where *A*_h_ is cross section of the piston, *A*_g_ is the section area of piston rod, *F*_*foil*_ is film friction force.

**Figure 6:**
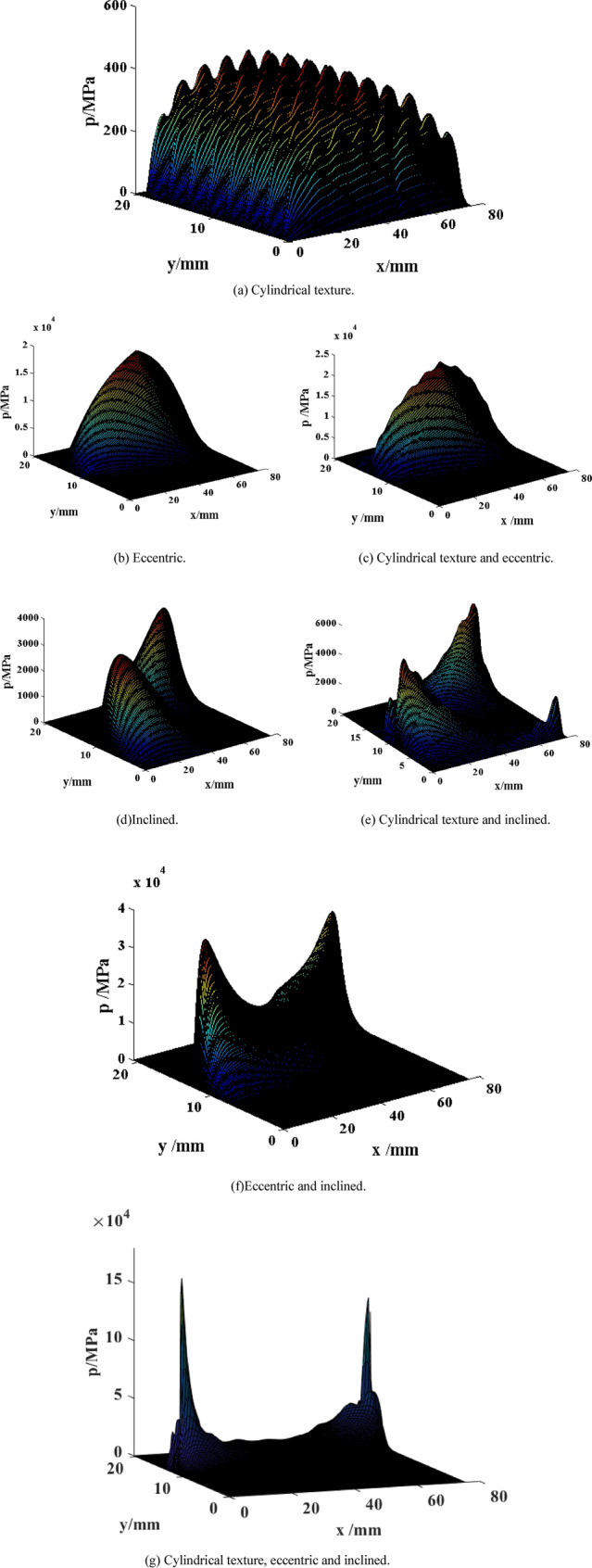
Distribution of pressure on the outer surface of the piston under different cases.

The oil film pressures on the outer surface of the piston are calculated under seven different cases of Fig. 5 by solving Reynolds Eq. (17). The pressures distribution on the outer surface of the piston can be obtained in Fig. 6. As a result, the change trend of oil film pressure *p* and that of actual oil film thickness *h* are consistent. The high oil film pressures between the piston and the cylinder are induced by increasing or decreasing oil film thickness (forming wedge gap) since wedge effect and extrusion effect. Thus, the friction force *F*_*foil*_ of oil film is generated when piston moves.

## Results and discussion

### The parameters of numerical simulation

The parameters of numerical simulation were provided in the Table 1 and as follow:

**Table 1:** Parameters of numerical simulation.

Parameters	Value
Flow coefficient of the constant orifice of piston assembly *C*_q_	0.82
The constant orifice diameter of piston assembly d_AT_/mm	0.65
Oil density *ρ*/kg/m^3^	900
Piston diameter *d*_h_/mm	24
Dynamic viscosity of the oil *μ*/Pa s	0.017
Axial width of piston *L*_*y*_/mm	20.5
Initial oil film thickness between piston and cylinder *h*_0_/mm	0.01
Flow coefficient of extension throttle orifice $\varepsilon_{fc}$	1
Total area of extension valve orifice *A*_*fc*_ /mm^2^	1.28
Outside radius of extension valve plate *r*_*bf*_ /mm	8
Notch radius of the extension valve plate *r*_*kf*_ /mm	5
Flow coefficient of the constant orifice of foot valve assembly $\varepsilon_{yc}$	1
Total area of the constant orifice of foot valve assembly *A*_*yc*_ /mm^2^	4.05
Outside radius of check valve plate *r*_*bb*_/mm	8
Notch radius of check valve plate *r*_*kb*_/mm	5
Thickness of valve plate *h*_*ffp*_/mm	1.2
Initial volume of reservoir chamber *V*_0_/m^3^	0.05 × 10^–3^
Initial pressure of reservoir chamber *P*_30_/MPa	1
Section area of piston rod *A*_g_/mm^2^	78.5

**Figure 7:**
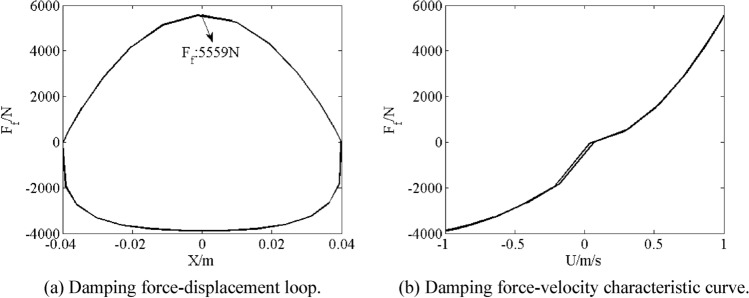
Simulation results of damping characteristic.

As shown in Fig. 7, damping force–displacement loop and damping force–velocity characteristic curve show effects of damping. Maximum damping force *F*_*f*_ is 5559 N.

### Effect of depth ratio *δ*

**Figure 8:**
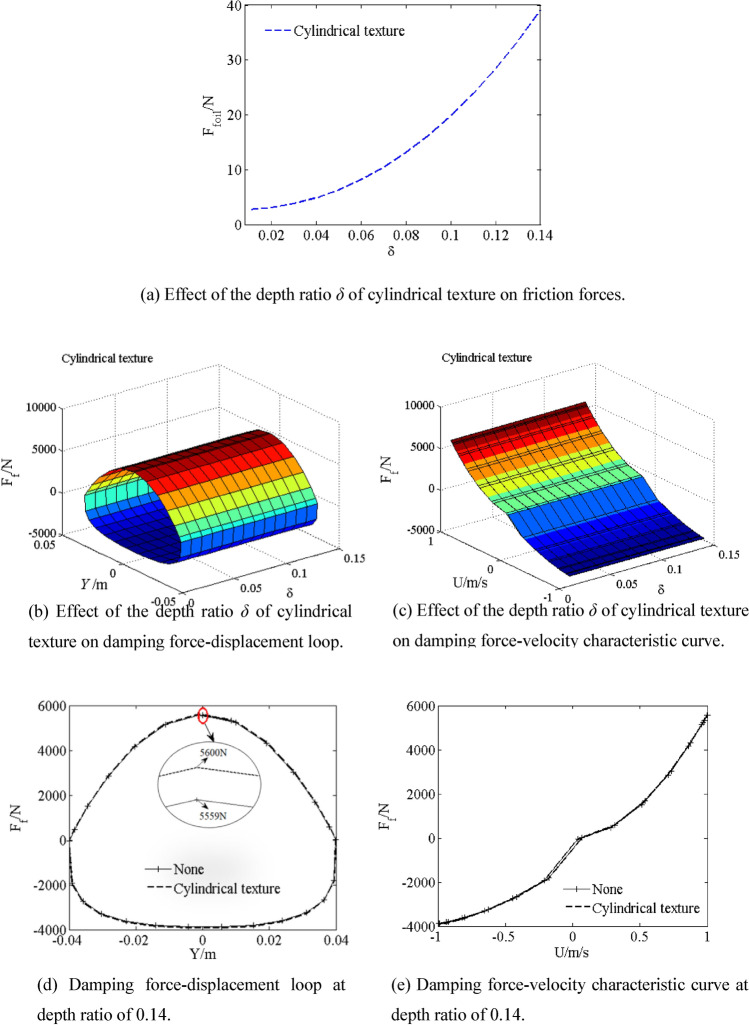
Effect of the depth ratio *δ* of cylindrical texture on damping characteristic.

The depth ratio *δ* is defined by ratio of texture depth *h*_p_ to initial oil film thickness *h*_0_ (*δ* = *h*_p_/*h*_0_). The friction forces *F*_*foil*_ of the cylindrical textured piston are shown by simulating with different depth ratio *δ* from 0.01 to 0.14 in Fig. 8a. The film friction force *F*_*foil*_ of the cylindrical texture increases with increasing depth ratio *δ*. The friction force *F*_*foil*_ of the cylindrical textured piston is 38 N at depth ratio *δ* of 0.14. The friction force *F*_*foil*_ and depth ratio *δ* shows parabolic curve relationship with increasing depth ratio *δ* from 0.01 to 0.14. However, as shown in Fig. 8b–e, the effect of friction force *F*_*foil*_ since the cylindrical texture on the damping characteristics can be neglected, which is consistent with the results in literature^17^. It also can be seen that the damping force *F*_*f*_ (5600 N) of the shock absorber with cylindrical textured piston increases by 0.74% when depth ratio *δ* increases to 0.14.

**Figure 9:**
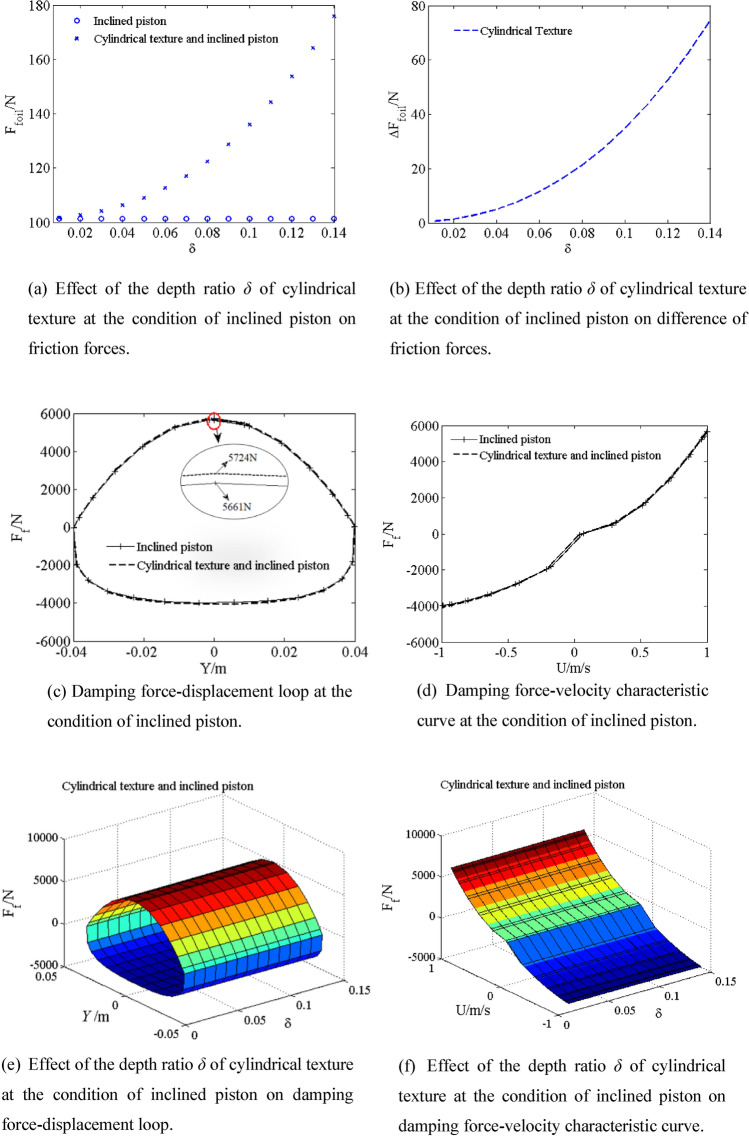
Effect of the depth ratio *δ* of cylindrical texture at the condition of inclined piston on damping characteristic.

The piston is inclined at an angle of 7.15 × 10^–4^ rad, the angle *β* between *OE*_2_ and *E*_1_ *E*_3_ is $\frac{\pi }{{2}}$ rad, the angle *φ* between *OE*_2_ and Z axis is $\frac{\pi }{{2}}$ rad. The friction force *F*_*foil*_ of the inclined piston and the cylindrical textured inclined piston are shown by simulating with different depth ratio δ from 0.01 to 0.14 in Fig. 9a, the difference Δ*F*_*foil*_ of friction force of the cylindrical texture inclined piston and inclined piston are shown in Fig. 9b. The friction force *F*_*foil*_ of the cylindrical texture inclined piston increases with increasing the depth ratio *δ* from 0.01 to 0.14. The friction force *F*_*foil*_ of the cylindrical textured inclined piston is higher than that of the inclined piston. The friction force *F*_*foil*_ and depth ratio *δ* shows parabolic curve relationship with increasing depth ratio *δ* from 0.01 to 0.14. Compared with friction force *F*_*foil*_ of inclined piston (101.2 N), when depth ratio *δ* is increased to 0.14, the difference Δ*F*_*foil*_ of friction force is 74.7 N, the friction force *F*_*foil*_ of the cylindrical textured inclined piston (175.9 N) greatly increases by 73.81%. However, the effect of friction force since the cylindrical texture inclined piston on the damping characteristics can be neglected in Fig. 9c–f. Compared with the damping force *F*_*f*_ of inclined piston (5661 N), when depth ratio *δ* is increased to 0.14, the damping force *F*_*f*_ of the cylindrical textured inclined piston (5724 N) greatly increases by 1.11%. As shown in Fig. 9e, the area of damping force–displacement loop slightly increases with increasing depth ratio *δ*. Thus, effect of the cylindrical textured at inclined piston condition on damping force can be neglected. Compared with the damping force F_*f*_ of piston (5559 N), when depth ratio δ is increased to 0.14 and inclined angle is 7.15 × 10^–4^ rad, the damping force *F*_*f*_ of the cylindrical textured inclined piston (5724 N) greatly increases by 3.02%.

**Figure 10:**
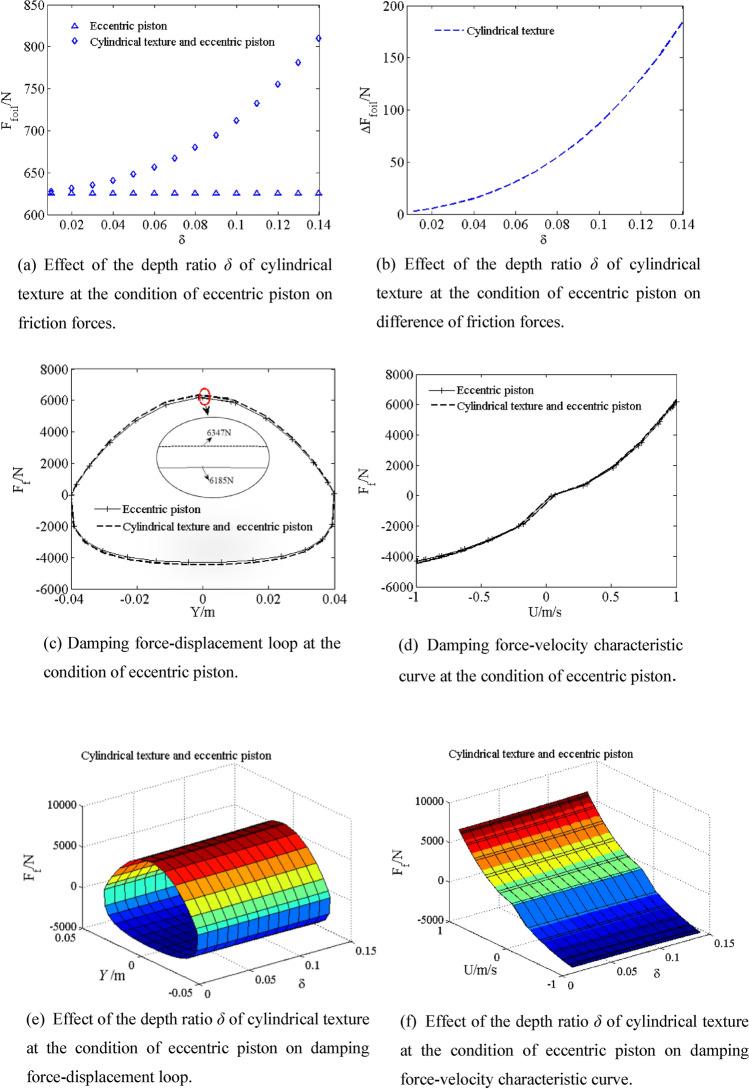
Effect of the depth ratio *δ* of cylindrical texture at the condition of eccentric piston on damping characteristic.

Piston is eccentric, the eccentricity *e* of the central section of the piston is 0.6h_0_. The friction force *F*_*foil*_ of the eccentric piston and the cylindrical textured eccentric piston are shown by simulating with depth ratio *δ* from 0.01 to 0.14 in Fig. 10a, the difference Δ*F*_*foil*_ of friction force of the cylindrical texture eccentric piston and eccentric piston is shown in Fig. 10b. The friction force *F*_*foil*_ of the cylindrical texture eccentric piston increases with increasing depth ratio *δ* from 0.01 to 0.14. The friction force *F*_*foil*_ and depth ratio *δ* shows parabolic curve relationship with increasing depth ratio *δ* from 0.01 to 0.14. Compared with friction force *F*_*foil*_ of eccentric piston (625.8 N), when depth ratio *δ* is increased to 0.14, the friction force *F*_*foil*_ of the cylindrical textured eccentric piston (810.3 N) greatly increases by 29.48%. In Fig. 10c–f, compared with the damping force *F*_*f*_ of eccentric piston (6185 N), when depth ratio *δ* is increased to 0.14, the damping force *F*_*f*_ the cylindrical textured eccentric piston (6347 N) greatly increases by 0.27%. As shown in Fig. 10e, the area of damping force–displacement loop slightly increases with increasing depth ratio *δ*. Thus, effect of the cylindrical textured at eccentric piston condition on damping force can be neglected. Compared with the damping force F_*f*_ of piston (5559 N), when depth ratio δ is increased to 0.14 and the eccentricity *e* is 0.6h_0_, the damping force *F*_*f*_ of the cylindrical textured eccentric piston (6185 N) greatly increases by 11.26%. As a result, the friction force since the cylindrical texture eccentric piston has great effects on damping characteristics, as shown in Figs. 7c and 10c,d.

**Figure 11:**
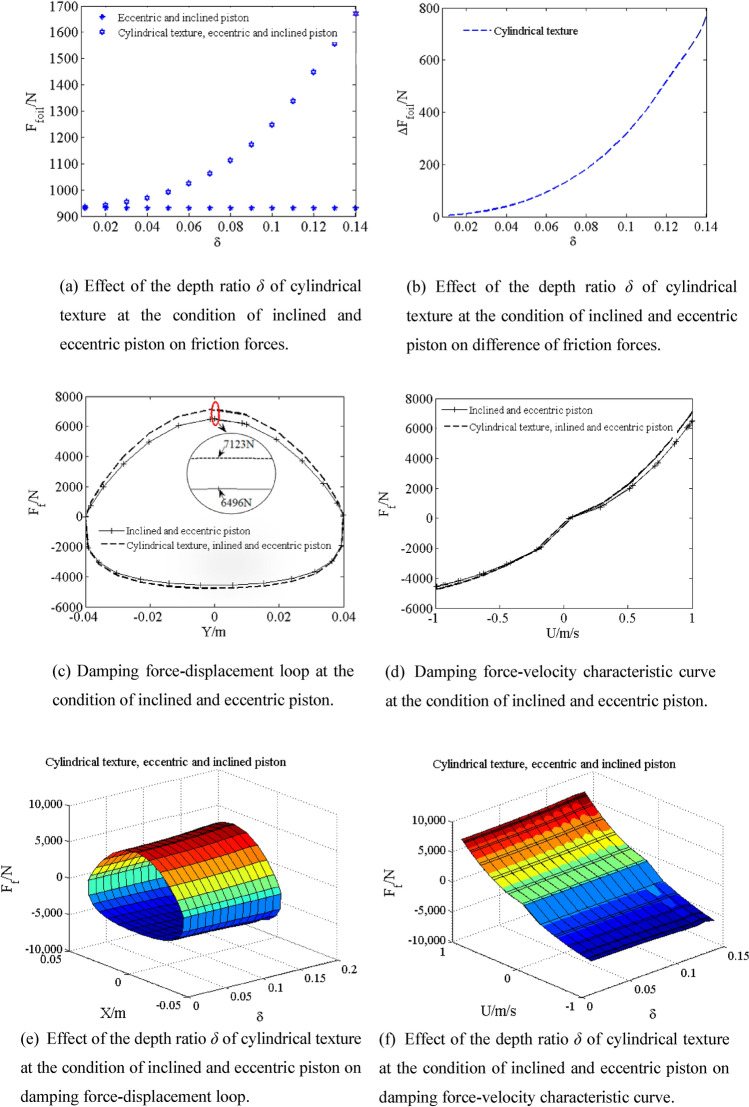
Effect of the depth ratio *δ* of cylindrical texture at the condition of inclined and eccentric piston on damping characteristic.

Piston is inclined and eccentric. The friction force *F*_*foil*_ of the inclined and eccentric piston and the cylindrical textured inclined and eccentric piston are shown by simulating with the depth ratio *δ* from 0.01 to 0.14 in Fig. 11a. The difference Δ*F*_*foil*_ of friction force of the cylindrical texture inclined and eccentric piston and inclined and eccentric piston is shown in Fig. 11b. The friction force *F*_*foil*_ of the cylindrical texture inclined and eccentric piston increases with increasing the depth ratio *δ* from 0.01 to 0.14. The friction force *F*_*foil*_ and depth ratio *δ* shows parabolic curve relationship with increasing depth ratio *δ* from 0.01 to 0.14. Compared with friction force *F*_*foil*_ of inclined and eccentric piston (930.3 N), when the depth ratio *δ* is increased to 0.14, the friction force *F*_*foil*_ of the cylindrical textured inclined and eccentric piston (1682 N) greatly increases by 80.8%. As a result, the cylindrical texture under inclined and eccentric piston condition has great effects on the friction force *F*_*foil*_. As shown in Fig. 11c,d, Compared with the damping force *F*_*f*_ of inclined and eccentric piston (6496 N), when the depth ratio *δ* is increased to 0.14, the damping force *F*_*f*_ the cylindrical textured inclined and eccentric piston (7123 N) greatly increases by 9.65%. Therefore, it is worth noting that the piston eccentricity plus inclination with cylindrical texture has great effect on damping force. As shown in Fig. 11e,f, the area of damping force–displacement loop increases with increasing depth ratio *δ*. As shown in Figs. 7 and 11c,d, taking the damping force F_f_ of piston (5559 N) as the baseline value, when depth ratio δ is increased to 0.14 and the eccentricity *e* is 0.6h_0_, the damping force *F*_*f*_ of the cylindrical textured inclined and eccentric piston (7123 N) greatly increases by 28.13%. As shown in Figs. 9c,d, 10c,d and 11c,d, it can be concluded that, compared with inclined piston with cylindrical texture or eccentric piston with cylindrical texture, the damping force *F*_*f*_ of the inclined and eccentric pistion with cylindrical textured not only is greater, but also is greater than combination of two situations.

### Effect of area ratios S_p_

**Figure 12:**
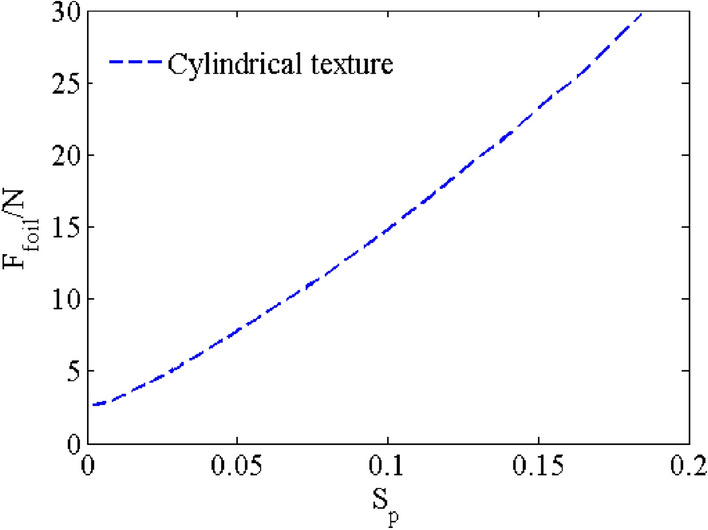
Effect of the area ratio S_p_ of cylindrical texture on friction forces.

The area ratio is defined as $S_{p} = \frac{{4n\pi R_{p}^{2} }}{{{2}\pi RL_{y} }}$,where *n* is the number of textures (*n* = 16), *R*_p_ is radius of cylinder texture, *R* is radius of piston. The friction forces *F*_*foil*_ of the cylindrical textured piston is shown by simulating with different area ratios S_p_ from 0.0003 to 0.18 in Fig. 12. The film friction force *F*_*foil*_ of the cylindrical texture increases with increasing area ratios S_p_ from 0.0003 to 0.18, and the friction force *F*_*foil*_ of the cylindrical textured piston is 23.8 N at area ratios S_p_ of 0.18. *F*_*foil*_-S_p_ curve is approximately linear. However, the effect of friction force since the cylindrical texture on the damping characteristics can be neglected, which is consistent with the results in literature^17^.

**Figure 13:**
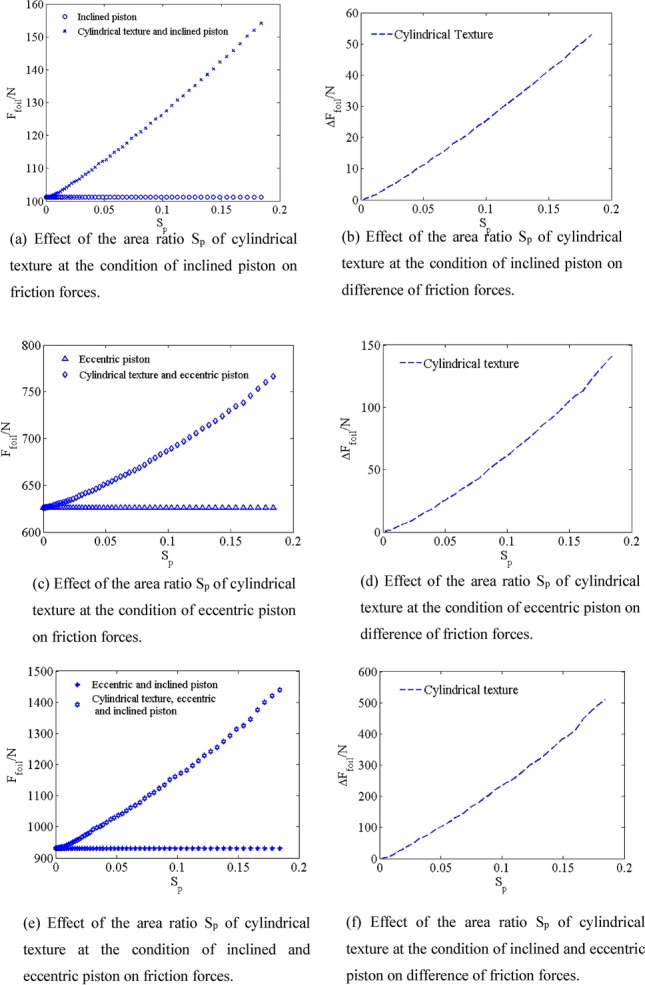
Effect of the area ratio S_p_ of cylindrical texture at different conditions on friction forces.

The piston is inclined at an angle of 7.15 × 10^–4^ rad, the angle *β* between *OE*_2_ and *E*_1_ *E*_3_ is $\frac{\pi }{{2}}$ rad, the angle *φ* between *OE*_2_ and Z axis is $\frac{\pi }{{2}}$ rad. The friction force *F*_*foil*_ of the inclined piston and the cylindrical textured inclined piston are shown by simulating with different area ratios S_p_ from 0.0003 to 0.18 in Fig. 13a, the difference Δ*F*_*foil*_ of friction force of the inclined piston and the cylindrical texture inclined piston is shown in Fig. 13b. The friction force *F*_*foil*_ of the cylindrical texture inclined piston increases with increasing area ratios S_p_ from 0.0003 to 0.18, and the friction force *F*_*foil*_ of the cylindrical textured inclined piston is higher than that of the inclined piston. *F*_*foil*_-S_p_ curves are approximately linear. Compared with friction force *F*_*foil*_ of inclined piston (101.2 N), when area ratios S_p_ is increased to 0.18, the friction force *F*_*foil*_ of the cylindrical textured inclined piston (154.1 N) greatly increases by 52.27%. The cylindrical textured at inclined piston condition has great effects on friction force. When piston is eccentric, the eccentricity *e* of the central section of the piston is 0.6h_0_. The friction force *F*_*foil*_ of the eccentric piston and the cylindrical textured eccentric piston are shown by simulating with different area ratios S_p_ from 0.0003 to 0.18 in Fig. 13c, the difference Δ*F*_*foil*_ of friction force of the cylindrical texture eccentric piston and eccentric piston is shown in Fig. 13d. The friction force *F*_*foil*_ of the cylindrical texture eccentric piston increases with increasing area ratios S_p_ from 0.0003 to 0.18. *F*_*foil*_- S_p_ curves are approximately linear. Compared with friction force *F*_*foil*_ of eccentric piston (625.8 N), when area ratios S_p_ is increased to 0.18, the friction force *F*_*foil*_ of the cylindrical textured eccentric piston (766.4 N) greatly increases by 22.47%. Thus, the cylindrical textured at eccentric piston condition has great effects on friction force. When piston is inclined and eccentric, the friction force *F*_*foil*_ of the inclined and eccentric piston and the cylindrical textured inclined and eccentric piston are shown by simulating with different area ratios S_p_ from 0.0003 to 0.18 in Fig. 13e, the difference Δ*F*_*foil*_ of friction force of the cylindrical texture inclined and eccentric piston and inclined and eccentric piston is shown in Fig. 13f. The friction force *F*_*foil*_ of the cylindrical texture inclined and eccentric piston increases with increasing area ratios S_p_ from 0.0003 to 0.18. *F*_*foil*_- S_p_ curves are approximately linear. Compared with friction force *F*_*foil*_ of inclined and eccentric piston (930.3 N), when area ratios S_p_ is increased to 0.18, the friction force *F*_*foil*_ of the cylindrical textured inclined and eccentric piston (1439 N) greatly increases by 54.68%. As a result, the cylindrical texture at inclined and eccentric piston condition has great effects on the friction force *F*_*foil*_.

As a result, the results of this study might provide a new insight for the design of hydraulic shock absorber and investigation of vehicle system dynamics. Since the upstream and downstream of the piston may change frequently due to the reciprocating motion of the piston, it is necessary to consider the flow conservation law of the lubricant including the cavitation region. Cavitation has great effects on damping characteristics. The disadvantages of these theoretical studies also include the lack of changes in thermophysical properties, primarily the coefficient of dynamic viscosity, from temperature, as well as the determination of temperature changes in the working cavities of the shock absorber. However, it is regrettable that their effects are ignored in this research. The future works should be committed to develop a detail model including oil temperature and cavitation to analyzed more detailed damping characteristics.

## Conclusion

In present work, a more detailed mathematical model was developed to estimate the effects of piston texture on damping characteristics of shock absorbers, which taking account of piston slight inclination and eccentricity, and depth ratio *δ* of piston texture and area ratio S_p_ of piston texture on friction force and damping characteristics at piston slight inclination and eccentricity conditions were analyzed in detail. The conclusions of current work can be drawn as follow:

1. Based on the mathematical models of reservoir and compression stroke coupled with Reynolds equation, a new damping force model is developed. The mathematical models of piston texture, piston slight inclination, piston eccentricity and combinations of three cases in turn are developed.
2. The cylindrical texture of piston has great effects on the friction force at three different conditions. The friction force of piston increases parabolically with increasing depth ratio *δ* of piston texture, and that of piston increases linearly with increasing area ratio S_p_ of piston texture.
3. The cylindrical texture of piston has little effects on damping characteristics at specific structural parameters condition when piston normal operates. The cylindrical texture of piston has great effects on damping characteristics at piston eccentricity and incline condition. The damping force *F*_*f*_ of the cylindrical textured inclined and eccentric piston might greatly increases under certain parameters.
